# Evaluation of cardiovascular disease burden and therapeutic goal attainment in US adults with chronic kidney disease: an analysis of national health and nutritional examination survey data, 2001–2010

**DOI:** 10.1186/1471-2369-14-132

**Published:** 2013-06-27

**Authors:** Andreas Kuznik, Jack Mardekian, Lisa Tarasenko

**Affiliations:** 1 Pfizer Inc, New York, NY, USA; 2 Global Health Economics and Outcomes Research, Pfizer Inc, 235 E 42nd St, New York, NY 10017, USA

**Keywords:** Chronic Kidney Disease, Low-density Lipoprotein Cholesterol, Blood Pressure, Cardiovascular Risk Factors, Goal Attainment

## Abstract

**Background:**

For chronic kidney disease (CKD) patients, national treatment guidelines recommend a low-density lipoprotein cholesterol (LDL-C) goal <100 mg/dL and blood pressure (BP) target <130/80 mmHg. This analysis assessed the current status of cardiovascular (CV) risk factor treatment and control in US adults with CKD.

**Methods:**

Weighted prevalence estimates of CV-related comorbidities, utilization of lipid- and BP-lowering agents, and LDL-C and BP goal attainment in US adults with CKD were assessed among 9,915 men and nonpregnant women aged ≥20 years identified from the fasting subsample of the 2001–2010 National Health and Nutritional Examination Survey (NHANES). Analyses were performed using SAS survey procedures that consider the complex, multistage, probability sampling design of NHANES. All estimates were standardized to the 2008 US adult population (≥20 years). Data were stratified by CKD stage based on presence of albuminuria and estimated glomerular filtration rate (eGFR), calculated using the Chronic Kidney Disease Epidemiology Collaboration (CKD-EPI) equation. Stage 3 CKD was subdivided into 3a (eGFR 45–59 mL/min/1.73 m^2^) and 3b (eGFR 30–44 mL/min/1.73 m^2^); Stage 5 CKD and dialysis recipients were excluded.

**Results:**

Of the 9,915 NHANES participants identified for analysis, 1,428 had CKD (Stage 1–4), corresponding to a prevalence estimate for US adults aged ≥20 years of 10.2%. Prevalence of CV-related comorbidities increased markedly with CKD stage, with a ~6–12-fold increase in cardiovascular disease, coronary heart disease (CHD), stroke and congestive heart failure between CKD Stage 1 and 4; prevalence of diabetes, hyperlipidemia and hypertension increased by ~1.2–1.6-fold. Use of lipid-lowering agents increased with CKD stage, from 18.1% (Stage 1) to 44.8% (Stage 4). LDL-C goal attainment increased from 35.8% (Stage 1) to 52.8% (Stage 3b), but decreased in Stage 4 (50.7%). BP goal attainment decreased between Stage 1 and 4 (from 49.5% to 30.2%), despite increased use of antihypertensives (from 30.2% to 78.9%).

**Conclusions:**

Individuals with CKD have a high prevalence of CV-related comorbidities. However, attainment of LDL-C or BP goals was low regardless of disease stage. These findings highlight the potential for intensive risk factor modification to maximize CV event reduction in CKD patients at high risk for CHD.

## Background

Chronic kidney disease (CKD) is stratified into 5 distinct stages (Stage 1–5) based on the presence of persistent kidney damage and/or decreased glomerular filtration rate (GFR) [1,2], and affects an estimated 26 million adults in the United States [3]. It is well documented that individuals with CKD are at very high risk of cardiovascular (CV) morbidity and mortality [1,4-6]. This increased risk is highlighted by the observation that CKD patients are more likely to die of cardiovascular disease (CVD) than progress to end-stage renal disease (ESRD) [7-9]. Hence, CKD is considered a coronary heart disease (CHD) risk equivalent [1,5,10].

Traditional risk factors such as dyslipidemia and hypertension are major determinants of CVD in those with CKD [4,11,12]. Both are prevalent among patients with Stage 1–4 CKD [13,14]: depending on the patient population, up to 85% have a low-density lipoprotein cholesterol (LDL-C) >130 mg/dL and up to 95% have a blood pressure (BP) ≥140/90 mmHg. *Post-hoc* analyses of CV outcomes trials have indicated that pharmacological treatment of dyslipidemia and hypertension reduces the risk of CV events in patients with CKD [15-20]. While the renoprotective effects of antihypertensive therapy in CKD are well-documented [12], recent data suggest that the pleiotropic effects of statins may also include the preservation of renal function [17,21-23]. As such, aggressive control of such modifiable CV risk factors is particularly important in this high-risk population.

Current national treatment guidelines for patients with CKD recommend an LDL-C goal of <100 mg/dL and a BP goal of <130/80 mmHg [11,12]. With respect to lipid-lowering therapy, treatment recommendations advocate the use of statins in addition to lifestyle modification to improve lipid profiles. Using National Health and Nutritional Examination Survey (NHANES) data covering the period from 2001 to 2010, this analysis assessed (1) the prevalence of CV-related comorbidities and CV risk factors, (2) the utilization of lipid-lowering and BP-lowering agents, and (3) rates of LDL-C or BP goal attainment in US adults stratified by CKD stage. A time-trend analysis of lipid and BP treatment and control in US adults with CKD was also conducted to assess linear trends in CV risk factor management over the five 2-year NHANES study cycles between 2001 and 2010.

## Methods

### Study design

NHANES is conducted by the National Center of Health Statistics, Centers for Disease Control and Prevention, as a cross-sectional, stratified, multistage probability sample survey of the US civilian, noninstitutionalized population [24,25]. NHANES data are derived from direct interviews regarding medical history, medication use and diet, as well as clinical examinations performed at mobile examination centers (including BP measurements) and laboratory tests (including blood and urine biochemistries).

From 1999, NHANES became a continuous survey, with no break between study cycles, and data are released in 2-year increments; this analysis used pooled data from the 5 most recent study cycles: 2001–2002, 2003–2004, 2005–2006, 2007–2008 and 2009–2010. Data from the 2001–2002 study cycle were included in this analysis to enable the assessment of linear trends in CV risk factor treatment and control before and after the release of current lipid and BP treatment guidelines for patients with CKD in 2003 and 2004, respectively [11,12]. NHANES 2001–2010 received approval from the National Center for Health Statistics research ethics review board, and written informed consent was obtained from all NHANES participants [24].

### Sample population

From the total 2001–2010 NHANES population of 52,195 participants, after excluding participants <20 years of age (n=24,611), participants that did not attend the mobile examination center (n=1,276), participants without fasting laboratory measurements (15,162), pregnant women (n=407), participants with missing lipid or BP data (n=788) and participants with Stage 5 CKD (n=36), a sample population of 9,915 participants was identified for analysis. This analysis was restricted to the fasting subsample of NHANES to enable the identification of participants with diabetes and hyperlipidemia, the definitions of which require valid fasting plasma glucose and LDL-C levels, respectively (described in further detail below). NHANES participants are randomly selected for inclusion in the fasting subsample and instructed to fast for 8 to <24 hours prior to blood specimens being taken for laboratory testing [25].

Data were stratified by CKD stage, categorized according to the presence of kidney damage (based on albuminuria) and level of decline in kidney function (based on estimated glomerular filtration rate [eGFR]). Albuminuria was defined as a urinary albumin–creatinine ratio of ≥30 mg/g. eGFR was calculated from serum creatinine concentration using the Chronic Kidney Disease Epidemiology Collaboration (CKD-EPI) equation [26]: eGFR = 141 × min(SCr/κ, 1)^α^ × max(SCr/κ, 1)^-1.209^ × 0.993^Age^ × 1.018 if female × 1.159 if black, where SCr is serum creatinine, κ is 0.7 for females and 0.9 for males, α is -0.329 for females and -0.411 for males, min indicates the minimum of SCr/κ or 1, and max indicates the maximum of SCr/κ or 1. CKD staging used a modification of National Kidney Foundation (NKF) criteria [1]: Stage 1, albuminuria with an eGFR ≥90 mL/min/1.73 m^2^; Stage 2, albuminuria with an eGFR 60–89 mL/min/1.73 m^2^; Stage 3 was subdivided into Stage 3a, an eGFR 45–59 mL/min/1.73 m^2^, and Stage 3b, an eGFR 30–44 mL/min/1.73 m^2^; and Stage 4, an eGFR 15–29 mL/min/1.73 m^2^. Individuals with Stage 5 CKD (<15 mL/min/1.73 m^2^) and those on dialysis were not included in the study due to the likelihood of confounding from the small number of individuals within these groups.

### Data collection and laboratory measurements

All disease history and drug utilization was self-reported based on the NHANES questionnaire. CVD was a composite of self-reported CHD, stroke or congestive heart failure. CHD was identified by self-report of CHD, angina or myocardial infarction (MI). Presence of diagnosed or undiagnosed diabetes was identified by self-report of diabetes, self-reported use of insulin or oral medications for diabetes, or a fasting plasma glucose ≥126 mg/dL. Hyperlipidemia was defined as fasting levels of LDL-C above the specific goal for each CHD risk category designated in the National Cholesterol Education Program Adult Treatment Panel III (NCEP ATP III) guidelines [27] (LDL-C level ≥160 mg/dL for individuals with ≤1 CHD risk factor, ≥130 mg/dL for individuals with ≥2 CHD risk factors, and ≥100 mg/dL for individuals with a history of CHD or CHD risk equivalents), or self-reported use of lipid-lowering agents (including statins, fibric acid derivatives, bile acid sequestrants, cholesterol absorption inhibitors and other antihyperlipidemic agents). CHD risk factors included cigarette smoking, hypertension (BP ≥140/90 mmHg or on antihypertensive medication), low levels of high-density lipoprotein cholesterol (HDL-C; <40 mg/dL), family history of premature CHD (male first-degree relative <55 years; female first-degree relative <65 years), and older age (men ≥45 years; women ≥55 years). CHD risk equivalents included diabetes and 2 or more risk factors conferring a 10-year risk for CHD >20%; information on non-coronary forms of atherosclerotic disease (peripheral arterial disease, abdominal aortic aneurysm, and symptomatic carotid artery disease; also considered CHD risk equivalents) was not available in NHANES. BP measurements in NHANES were performed 3–4 times manually with a mercury sphygmomanometer according to a standard protocol [25]. The first reading was excluded and the remaining readings were used to compute average BP. Hypertension was defined as an average BP >130 mmHg systolic or >80 mmHg diastolic, or self-reported use of antihypertensive agents (including β-blockers, calcium channel blockers, diuretics, angiotensin-converting enzyme inhibitors, angiotensin receptor blockers and other BP-lowering agents).

Methods for quantifying measures of kidney damage and kidney function have been described elsewhere [25]. Briefly, urinary albumin was measured using a solid-phase fluorescent immunoassay. Urinary creatinine was measured by one of two methods: a Jaffé rate (kinetic alkaline picrate) method (prior to 2007) and an enzymatic (creatinase) method (from 2007 on). Serum creatinine was measured using the Jaffé rate method. Methods for determining blood lipid levels in NHANES have been described previously [25]. Briefly, total cholesterol was measured enzymatically on the basis of hydrogen peroxide generation. In 2001–2002, high-density lipoprotein cholesterol (HDL-C) was measured using two methods, heparin–manganese precipitation and a direct immunoassay, depending on the participant age and amount of specimen. From 2003, all HDL-C measurements used the direct immunoassay method. Triglyceride levels were measured after hydrolysis to glycerol. LDL-C levels were calculated from measured values of total cholesterol, triglycerides (≤400 mg/dL) and HDL-C according to the Friedewald calculation [25]. Plasma glucose was measured using a modified hexokinase enzymatic method [25].

### Definition of treatment goals

Participants were classified as meeting current recommendations on LDL-C or BP treatment goals for patients with CKD [11,12] if their fasting LDL-C level was <100 mg/dL or their BP was ≤130/80 mmHg. A sensitivity analysis to investigate the effect of increasing the threshold for BP goal attainment to ≤140/90 mmHg was also performed. In participants with CKD and concomitant CVD, or CKD and concomitant diabetes, attainment of the optional LDL-C goal of <70 mg/dL [28-30] was also examined.

### Statistical methods

Statistical analyses were performed using survey analysis procedures available in SAS software version 9.22 (SAS Institute Inc., Cary, North Carolina) that take into account the complex sampling scheme of NHANES, and used sampling weights to account for differential probabilities of sample selection and non-response. The fasting sampling weights of the 9,915 participants included in the analysis were adjusted to the July 2008 US census population ≥20 years of age (n=221,419,638). Each 2-year fasting sample weight within an NHANES 2-year study cycle was multiplied by the 2008 US census count and divided by the 2-year weighted total sample count from the analysis data set of persons in the 2-year study cycle. The population sizes for each study cycle were 180,717,445 for 2001–2002; 184,340,382 for 2003–2004; 190,068,016 for 2005–2006; 201,486,048 for 2007–2008; and 203,258,815 for 2009–2010. For example, the fasting sampling weight for each study participant from 2001–2002 was multiplied by 221,419,638/180,717,445; the fasting sampling weight for each study participant from 2003–2004 was multiplied by 221,419,638/184,340,382; and so on for all 5 study periods. Demographic and clinical characteristics of the 2001–2010 NHANES participants with and without CKD were calculated using SURVEYFREQ. Rao-Scott chi-square *P* values for categorical variables were obtained using SURVEYFREQ. Between-cohort *P* values for continuous variables were obtained using SURVEYREG. Each continuous outcome was regressed on the indicator variable CKD=1 or No CKD=0, and a contrast statement was used to generate the between-cohort *P* value. Estimated population prevalences were calculated using SURVEYFREQ and stratified by CKD stage. *P* values for Stage 1 versus Stage 4 CKD were obtained using SURVEYLOGISTIC. Each outcome was regressed on the 5-level class variable CKD stage (Stage 1, 2, 3a, 3b or 4), and a contrast statement was used to generate the Stage 1 versus Stage 4 *P* value. Linear trends for utilization of lipid- and BP-lowering agents, and rates of LDL-C or BP goal attainment, over the five 2-year survey cycles were also assessed using SURVEYLOGISTIC, including time as a continuous variable. Statistical tests were 2-sided and a *P*-value <0.05 was considered statistically significant.

## Results

### Prevalence of CKD

Of the 9,915 NHANES participants identified from the 2001–2010 survey period, 1,428 had CKD (Stage 1–4), corresponding to a prevalence estimate for US adults aged ≥20 years of 10.2%. Among those persons with CKD, around half (49.1%; n=746) had Stage 3 CKD, comprising of 36.5% (n=545) with Stage 3a and 12.5% (n=201) with Stage 3b CKD; 3.7% (n=60) had Stage 4 CKD.

### Characteristics of persons with and without CKD

The demographic and clinical characteristics of the US adult population with CKD (Stage 1–4), based on NHANES participants from the 2001–2010 survey period, are shown in Table 1. Those with CKD (mean ± standard error [SE] eGFR: 68.4 ± 1.0 mL/min/1.73 m^2^) were older; were more likely to be female and of non-Hispanic white origin; had a higher body mass index; had higher systolic but lower diastolic BP; had higher triglyceride but lower LDL-C levels; and were more likely to be taking medication for diabetes when compared with persons without CKD (mean ± SE eGFR: 97.9 ± 0.4 mL/min/1.73 m^2^). A comparison of the demographic and clinical characteristics of the US adult population with CKD stratified by LDL-C and BP goal attainment status is provided in Additional file 1: Table S1. Those at LDL-C goal had a lower eGFR; had lower BP and lipid levels; had higher levels of medication use; and were more likely to have a history of CVD and diabetes but less likely to have hyperlipidemia compared with those not at LDL-C goal. Similar results were obtained for the BP goal and dual goal (LDL-C and BP) cohorts, with the exception that those at BP goal were younger and had a higher eGFR but lower body mass index compared with those not at BP goal.

**Table 1:** Population characteristics of US adults ≥20 years of age with and without CKD stage 1–4* based on NHANES 2001–2010 survey participants

**Characteristic**	**With CKD**	**Without CKD**	***P*†**
	**(n=1,428)**	**(n=8,487)**	
Age at screening (years)	64.2 (0.7)	44.5 (0.3)	<0.001
Male (%)	41.7 (1.5)	49.5 (0.5)	<0.001
Race/ethnicity (%)			0.001
Non-Hispanic white	75.4 (2.1)	71.2 (1.4)	
Non-Hispanic black	11.6 (1.1)	10.7 (0.8)	
Mexican American	5.7 (0.9)	8.0 (0.7)	
Other	7.3 (1.4)	10.1 (0.8)	
eGFR (mL/min/1.73 m^2^)	68.4 (1.0)	97.9 (0.4)	<0.001
Body mass index (kg/m^2^)	29.7 (0.3)	28.2 (0.1)	<0.001
	(n=1,372)	(n=8,398)	
Blood pressure (mmHg)			
Systolic	133.2 (0.8)	119.4 (0.2)	<0.001
Diastolic	67.5 (0.6)	70.2 (0.2)	<0.001
Lipids (mg/dL)			
Total cholesterol	194.3 (1.4)	196.4 (0.6)	0.1
LDL-C	111.4 (1.1)	117.6 (0.5)	<0.001
HDL-C	54.4(0.5)	54.0 (0.2)	0.6
Triglycerides	142.6 (2.4)	124.2 (1.0)	<0.001
Antidiabetic medication use (%)‡§	21.0 (1.4)	4.8 (0.2)	<0.001

Values are weighted estimates presented as percent (standard error) or mean (standard error). Estimates were standardized to the July 2008 US census population ≥20 years of age. Conversion factors for units: eGFR in mL/min/1.73 m^2^ to mL/s/1.73 m^2^, ×0.01667; total cholesterol, LDL-C and HDL-C in mg/dL to mmol/L, ×0.02586; triglycerides in mg/dL to mmol/L, ×0.01129; glucose in mg/dL to mmol/L, ×0.05551.

*CKD Stage 1–4 was identified by the presence of kidney damage (based on albuminuria) and level of decline in kidney function (based on eGFR), and staged using modified National Kidney Foundation criteria (see Methods).

†*P* values are for with versus without CKD; *P* value for race/ethnicity compares the distribution of race/ethnicity categories. Rao-Scott chi-square *P* values for categorical variables were obtained using the SAS procedure SURVEYFREQ; between-cohort *P* values for continuous variables were obtained using the SAS procedure SURVEYREG (see Methods).

‡All drug utilization was self-reported.

§Any antidiabetic agents including insulin and oral medications for diabetes.

### Prevalence of CV-related comorbidities and CV risk factors in persons with CKD

Overall, the prevalence of CV-related comorbidities and CV risk factors was higher in persons with versus those without CKD (all *P*<0.001; Table 2). The prevalence of CV-related comorbidities in persons with CKD was high: 19.6% had CHD; 10.3% stroke; and 9.7% congestive heart failure (CHF). CVD—a composite of CHD, stroke or CHF—was prevalent in 28.4% of those with CKD. The prevalence of CV risk factors in persons with CKD was also high: 31.5% had diabetes; 53.9% hyperlipidemia; and 76.1% hypertension. Between CKD Stage 1 and 4, there was a ~6–12-fold increase in the prevalence of CVD (from 9.0% to 51.0%; *P*<0.001), CHD (from 6.0% to 36.5%; *P*<0.001), stroke (from 2.5% to 30.3%; *P*<0.001) and CHF (from 3.2% to 27.8%; *P*<0.001) (Table 2). There was a ~1.2–1.6-fold increase in the prevalence of diabetes (from 36.0% to 44.7%; *P*=0.3), hyperlipidemia (from 45.5% to 67.8%; *P<*0.001) and hypertension (from 58.2% to 94.2%; *P*<0.001) between CKD Stage 1 and 4 (Table 2). Of note was the marked increase in disease burden between CKD Stage 3a and 3b for CVD (*P*<0.001), CHD (*P*=0.023), stroke (*P=*0.001), CHF (*P*<0.001) and diabetes (*P*=0.003) (Table 2).

**Table 2:** Prevalence of CV-related comorbidities and CV Risk Factors by CKD Stage in US adults ≥20 years of age with CKD Stage 1–4* based on 2001–2010 NHANES participants

**Variable**	**No CKD**	**All CKD**	**CKD Stage:**					***P*†**
	**(n=8,487)**	**(n=1,428)**	**1**	**2**	**3a**	**3b**	**4**	
			**(n=285)**	**(n=337)**	**(n=545)**	**(n=201)**	**(n=60)**	
Cardiovascular disease history (%)‡								
Cardiovascular disease§	6.0 (0.3)	28.4 (1.6)	9.0 (2.2)	31.2 (3.3)	30.2 (2.4)	49.4 (4.5)	51.0 (6.5)	<0.001
Coronary heart disease║	4.3 (0.3)	19.6 (1.3)	6.0 (1.9)	22.0 (2.8)	21.0 (2.1)	32.8 (4.5)	36.5 (5.7)	<0.001
Stroke	1.9 (0.2)	10.3 (1.1)	2.5 (0.8)	8.8 (1.8)	10.2 (1.4)	22.5 (4.3)	30.3 (7.1)	<0.001
Congestive heart failure	1.2 (0.1)	9.7 (0.9)	3.2 (1.1)	7.8 (1.8)	7.2 (1.1)	27.7 (3.4)	27.8 (4.9)	<0.001
Cardiovascular risk factors (%)‡								
Diabetes	8.1 (0.4)	31.5 (1.5)	36.0 (3.6)	32.4 (3.2)	24.4 (2.5)	38.3 (4.2)	44.7 (6.8)	0.3
Hyperlipidemia#	31.0 (0.6)	53.9 (1.5)	45.5 (3.3)	53.8 (3.1)	55.1 (2.6)	63.4 (4.5)	67.8 (5.5)	<0.001
Hypertension**	37.7 (0.8)	76.1 (1.5)	58.2 (3.9)	75.2 (3.6)	83.4 (2.3)	85.3 (2.4)	94.2 (2.7)	<0.001

Values are weighted estimates presented as percent (standard error). Estimates were standardized to the July 2008 US census population ≥20 years of age.

*CKD Stage 1–4 was identified by the presence of kidney damage (based on albuminuria) and level of decline in kidney function (based on eGFR), and staged using modified National Kidney Foundation criteria (see Methods).

†*P* values are for Stage 1 versus Stage 4 CKD, obtained using the SAS procedure SURVEYLOGISTIC; *P* values for No CKD versus All CKD are all <0.001, obtained using the SAS procedure SURVEYFREQ (see Methods).

‡All disease history and drug utilization was self-reported.

§Cardiovascular disease was a composite of self-reported CHD, stroke or CHF.

║Coronary heart disease was identified by self-report of CHD, angina or myocardial infarction.

¶Diabetes was identified by self-report, self-reported use of insulin or oral medications for diabetes, or fasting glucose ≥126 mg/dL.

#Hyperlipidemia was defined as LDL-C ≥160 mg/dL for individuals with ≤1 CHD risk factor, ≥130 mg/dL for individuals with ≥2 CHD risk factors, ≥100 mg/dL for individuals with CHD or CHD risk equivalents (eg, diabetes), or self-reported use of lipid-lowering agents.

**Hypertension was defined as an average BP >130 mmHg systolic or >80 mmHg diastolic, or self-reported use of antihypertensive agents.

### Lipid treatment and control in persons with CKD

Overall, the self-reported use of lipid-lowering agents was higher in persons with versus those without CKD (30.4% versus 11.8%; *P*<0.001; Table 3). The use of lipid-lowering agents increased with the degree of renal impairment, from 18.1% in those with CKD Stage 1 to 44.8% in those with CKD Stage 4 (*P*<0.001; Table 3). The overall proportion of persons with CKD achieving the LDL-C goal of <100 mg/dL was 40.0%. LDL-C goal attainment generally increased with CKD stage, from 35.8% in those with CKD Stage 1 to 52.8% in those with CKD Stage 3b, but decreased in those with CKD Stage 4 (to 50.7%; Table 3).

**Table 3:** Lipid and BP treatment and control rates by CKD stage in US adults ≥20 years of age with CKD Stage 1–4* based on 2001–2010 NHANES participants

**Variable**	**No CKD**	**All CKD**	**CKD Stage:**					***P*†**
	**(n=8,487)**	**(n=1,428)**	**1**	**2**	**3a**	**3b**	**4**	
			**(n=285)**	**(n=337)**	**(n=545)**	**(n=201)**	**(n=60)**	
Lipid treatment and control (%)‡								
Antihyperlipidemics§	11.8 (0.5)	30.4 (1.5)	18.1 (2.9)	31.4 (2.4)	33.0 (2.5)	40.4 (4.2)	44.8 (7.1)	<0.001
LDL-C <100 mg/dL	ND	40.0 (1.7)	35.8 (3.6)	38.2 (3.6)	38.4 (2.4)	52.8 (3.9)	50.7 (8.5)	0.135
BP treatment and control (%)‡								
Antihypertensives║	17.4 (0.6)	54.4 (1.6)	30.2 (3.2)	54.8 (3.7)	62.0 (2.5)	71.3 (3.4)	78.9 (5.8)	<0.001
BP ≤130/80 mmHg	ND	44.6 (1.8)	49.5 (4.0)	42.4 (3.7)	41.6 (2.9)	52.3 (4.3)	30.2 (6.2)	0.019

Values are weighted estimates presented as percent (standard error). Estimates were standardized to the July 2008 US census population ≥20 years of age. ND, not determined.

*CKD Stage 1–4 was identified by the presence of kidney damage (based on albuminuria) and level of decline in kidney function (based on eGFR), and staged using modified National Kidney Foundation criteria (see Methods).

†*P* values are for Stage 1 versus Stage 4 CKD, obtained using the SAS procedure SURVEYLOGISTIC; *P* values for No CKD versus All CKD are all <0.001, obtained using the SAS procedure SURVEYFREQ (see Methods).

‡All drug utilization was self-reported.

§Any lipid-lowering agents including statins, fibric acid derivatives, bile acid sequestrants, cholesterol absorption inhibitors and other antihyperlipidemic agents.

║Any antihypertensive agents including β-blockers, calcium channel blockers, diuretics, angiotensin-converting enzyme inhibitors, angiotensin receptor blockers and other BP-lowering agents.

### BP treatment and control in persons with CKD

Overall, the self-reported use of antihypertensive agents was higher in persons with versus those without CKD (54.4% versus 17.4%; *P*<0.001; Table 3). The use of BP-lowering medications increased between CKD Stage 1 and 4, from 30.2% in those with CKD Stage 1 to 78.9% in those with CKD Stage 4 (*P*<0.001; Table 3). However, despite the increased utilization of antihypertensives, BP goal attainment to ≤130/80 mmHg decreased between CKD Stage 1 and 4 (49.5% in Stage 1; 30.2% in Stage 4; *P*=0.019; Table 3). The overall proportion of persons with CKD achieving the BP goal of ≤130/80 mmHg was 44.6%. A sensitivity analysis increasing the threshold for BP goal attainment to ≤140/90 mmHg found this increased the proportion of persons with CKD classified as achieving BP goal by one-third, to 66.5%.

### Time-trend analysis of lipid and BP treatment and control rates in persons with CKD

**Figure 1:**
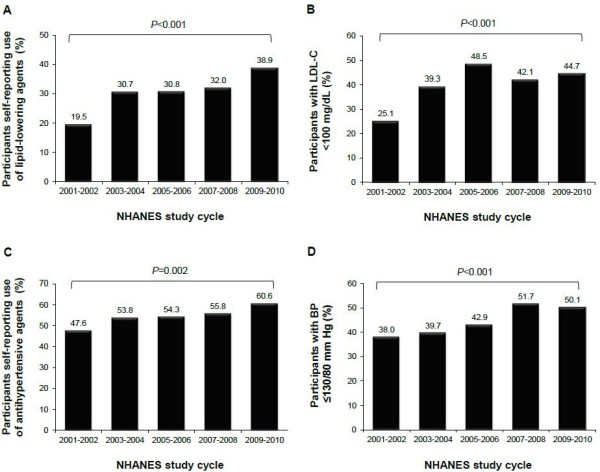
**Time-trend analysis of lipid and BP treatment and control rates in US adults ≥20 years of age with CKD Stage 1–4 based on 2001–2010 NHANES participants. A)** Proportion of persons with CKD with self-reported use of lipid-lowering agents. **B)** Proportion of persons with CKD at LDL-C goal <100 mg/dL. **C)** Proportion of persons with CKD with self-reported use of antihypertensive agents. **D)** Proportion of persons with CKD at BP goal ≤130/80 mmHg. *P* values are for trend over time, obtained using the SAS procedure SURVEYLOGISTIC (see Methods).

During the NHANES period examined, there was a significant increase in the self-reported use of lipid-lowering agents by persons with CKD, from 19.5% in 2001–2002 to 38.9% in 2009–2010 (*P*<0.001; Figure 1A). Over the same time frame, the proportion of the population achieving the LDL-C goal of <100 mg/dL also increased significantly, from 25.1% to 44.7% (*P*< 0.001; Figure 1B). Similarly, the self-reported use of antihypertensive agents by persons with CKD increased significantly over the 5 study cycles, from 47.6% in 2001–2002 to 60.6% in 2009–2010 (*P*=0.002; Figure 1C), as did achievement of the BP goal of ≤130/80 mmHg (38.0% in 2001–2002; 50.1% in 2009–2010; *P*<0.001; Figure 1D).

### Lipid and BP treatment and control in persons with CKD and concomitant CVD or diabetes

Table 4 shows lipid and BP treatment and control rates in persons with CKD and concomitant CVD or diabetes. For those with concomitant CKD and CVD, 50.7% reported using lipid-lowering agents and 52.8% had an LDL-C <100 mg/dL; 21.9% achieved the optional LDL-C goal of <70 mg/dL. BP treatment and control rates in this population were 72.7% and 46.3%, respectively (Table 4). For those with concomitant CKD and diabetes, 44.6% reported using lipid-lowering agents and 51.2% had an LDL-C <100 mg/dL; 17.5% achieved the optional LDL-C goal of <70 mg/dL. BP treatment and control rates in this population were 69.9% and 40.8%, respectively (Table 4).

**Table 4:** Lipid and BP treatment and control rates in US adults ≥20 years of age with CKD Stage 1–4* and concomitant CVD or diabetes based on 2001–2010 NHANES participants

**Variable**	With CKD Stage 1–4* and:	
	**Cardiovascular Disease†**	**Diabetes‡**
	**(n=434)**	**(n=501)**
Lipid treatment and control (%)§		
Antihyperlipidemics║	50.7 (2.3)	44.6 (2.7)
LDL-C <100 mg/dL	52.8 (2.3)	51.2 (2.5)
LDL-C <70 mg/dL	21.9 (2.0)	17.5 (2.3)
BP treatment and control (%)§		
Antihypertensives¶	72.7 (2.6)	69.9 (2.6)
BP ≤130/80 mmHg	46.3 (2.7)	40.8 (2.9)

Values are weighted estimates presented as percent (standard error). Estimates were standardized to the July 2008 US census population ≥20 years of age.

*CKD Stage 1–4 was identified by the presence of kidney damage (based on albuminuria) and level of decline in kidney function (based on eGFR), and staged using modified National Kidney Foundation criteria (see Methods).

†Cardiovascular disease was a composite of self-reported CHD, stroke or CHF.

‡Diabetes was identified by self-report, self-reported use of insulin or oral medications for diabetes, or fasting glucose ≥126 mg/dL.

§All drug utilization was self-reported.

║Any lipid-lowering agents including statins, fibric acid derivatives, bile acid sequestrants, cholesterol absorption inhibitors and other antihyperlipidemic agents.

¶Any antihypertensive agents including β-blockers, calcium channel blockers, diuretics, angiotensin-converting enzyme inhibitors, angiotensin receptor blockers and other BP-lowering agents.

### Dual lipid and BP goal attainment in persons with CKD, with or without concomitant CVD or diabetes

The overall proportion of persons with CKD who simultaneously achieved both an LDL-C <100 mg/dL and a BP ≤130/80 mmHg was 19.5%. Dual lipid and BP goal attainment was achieved in 28.1% of the population with concomitant CKD and CVD, and 24.9% of those with concomitant CKD and diabetes (LDL-C <100 mg/dL; BP ≤130/80 mmHg).

## Discussion

This analysis of NHANES data from 2001 to 2010 found that 10.2% of the US population ≥20 years of age had CKD Stage 1–4, representing an estimated 22.6 million Americans. CKD — particularly advanced CKD — was associated with a high prevalence of CV-related comorbidities: nearly 50% of US adults with CKD and an eGFR <45 mL/min/1.73m^2^ had concomitant CVD. Despite this, dyslipidemia was undertreated in this high-risk population (overall treatment rate: 30.4%). Furthermore, the proportions of individuals with CKD achieving recommended LDL-C or BP therapeutic goals were low, regardless of CKD stage. Although significant increases were seen in lipid and BP treatment and control rates over the 10-year survey period examined, ≤50% of persons with CKD Stage 1–4 are currently achieving the recommended LDL-C or BP therapeutic goals. Furthermore, this analysis revealed that despite the very high-risk combination of CKD and CVD, or CKD and diabetes, dyslipidemia was undertreated in these individuals, with only around half of the CKD population with concomitant CVD or diabetes receiving any form of lipid-lowering therapy and a similar proportion achieving an LDL-C <100 mg/dL; fewer still (~1 in 5) attained the optional goal of <70 mg/dL recommended for those individuals classified as being at very high risk of future CV events. Although a higher proportion of these very high-risk individuals received antihypertensive medications (~70%), still less than half were at BP goal ≤130/80 mmHg. Moreover, only ~1 in 5 people with CKD are achieving both the LDL-C and BP treatment targets simultaneously, with a slightly higher proportion (~1 in 4) of those considered to be at very high risk of future CV events (those with concomitant CVD or diabetes) at both LDL-C and BP goal. Together, these findings highlight an unmet medical need in CKD care and the potential for aggressive risk factor modification to maximize CV event reduction in CKD patients at high or very high risk for CHD.

Hyperlipidemia, defined in this analysis as levels of LDL-C above the specific goal for each NCEP ATP III CHD risk category [27] or self-reported use of lipid-lowering agents, was prevalent in the 2001–2010 US adult CKD population (53.9%). In the absence of hypertriglyceridemia, national treatment guidelines for patients with CKD identify LDL-C as the primary focus of lipid-lowering therapy and advocate the use of statins in addition to therapeutic lifestyle changes to improve lipid profiles [11]. Despite these recommendations, less than one-third of individuals with CKD reported using any lipid-lowering medication (30.4%), and just over one-third achieved the recommended LDL-C goal of <100 mg/dL (40.0%). Although it is not clear whether patients with very advanced renal impairment (ie, ESRD) or renal transplant recipients derive a CV benefit from statin therapy [31-33], meta-analyses [34,35] and *post-hoc* subgroup analyses from several large statin intervention trials [15-18,36] have indicated that aggressive treatment of dyslipidemia reduces the risk of CV events in patients with mild-to-moderate CKD. Moreover, the recent Study of Heart and Renal Protection (SHARP) — a prospective trial in 9,270 (3,023 dialysis and 6,247 predialysis) patients with CKD and no known history of MI or coronary revascularization — demonstrated that LDL-C reduction with ezetimibe/simvastatin combination therapy reduced the relative risk of a major atherosclerotic event (coronary death, MI, ischemic stroke, or revascularization) by 17% (RR, 0.83; 95% CI, 0.74–0.94; *P*=0.002) versus placebo, irrespective of the severity of renal disease [37]. The apparent discrepancy between trials such as 4D or AURORA and SHARP is currently the subject of much debate within the medical community, and has centered on differences in the primary end point and rates of specific CV outcomes across the 3 trials that might explain the lack of clear benefit in the earlier trials. For example, the primary end point in the 4D trial focused on cardiac death, and sudden cardiac death — less likely to be modifiable with statin therapy — accounted for >50% of the primary outcomes in this trial [31]. This is in stark contrast with the more specific, atherosclerotic end point, and the predominance of non-fatal atherosclerotic (and, hence, statin-modifiable) outcomes, in the SHARP trial [37]. Interestingly, heterogeneity analyses across 4 prospective statin trials in renal patients (SHARP, AURORA, 4D, and the Assessment of Lescol in Renal Transplantation [ALERT] trial) found a similar effect of LDL-C–lowering therapy on risk reduction for selected vascular outcomes, including nonfatal MI, nonfatal ischemic stroke, and coronary revascularization [37]. Given the high prevalence of CV-related comorbidities and CV risk factors observed in this analysis of NHANES participants with CKD, aggressive LDL-C–lowering therapy to an optional goal of <70 mg/dL may be warranted in those with multiple high-risk factors (eg, CKD plus diabetes or CKD plus established CHD), as suggested in current national treatment recommendations for patients at very high risk of future CV events [28-30]. However, we found that only ~20% of those individuals with CKD and concomitant CVD or diabetes were achieving this more-stringent LDL-C goal.

Hypertension (defined as an average BP >130 mmHg systolic or >80 mmHg diastolic, or self-reported use of antihypertensive agents) was also prevalent in the 2001–2010 US adult CKD population (76.1%). Current treatment recommendations indicate that, for most patients with CKD, the use of multiple antihypertensive agents will be required to achieve a BP goal of <130/80 mmHg and reduce CV risk [12]. Despite this, only around half of individuals with CKD reported using any antihypertensive medication (54.4%), and less than half achieved a BP goal of ≤130/80 mmHg (44.6%). A sensitivity analysis increasing the threshold for BP goal attainment to ≤140/90 mmHg increased the proportion of persons with CKD classified as achieving their BP goal to 66.5%. The lack of conclusive evidence from randomized controlled trials as to the CV benefit of strict versus standard BP control [38] has sparked intensive debate on the subject of an appropriate BP goal for patients with CKD. However, the observation from our analysis that over half of all US adults with CKD are not meeting the current BP treatment goal of ≤130/80 mmHg, and one-third are not meeting the less-stringent target of ≤140/90 mmHg, indicates that suboptimal management of hypertension persists in this high-risk population.

The restriction of this analysis to the fasting subsample of NHANES due to the requirement of valid glucose and LDL-C measurements may explain the somewhat counterintuitive fall in prevalence of CKD Stage 1–4 observed between this analysis of NHANES 2001–2010 data (10.2%; 22.6 million US adults; standardized to the 2008 US adult population) and an earlier analysis of NHANES 1999–2004 data (13.1%; 26.3 million US adults; standardized to the 2000 US adult population) [3], as the prevalence estimates presented here were based on around three-quarters of the number of NHANES participants as the previous study (n=9,915 versus n=13,233, respectively). Also, the use of the CKD-EPI equation in this analysis versus the Modification of Diet in Renal Disease (MDRD) Study equation in the earlier analysis [3] may have also contributed to the reduced estimated CKD prevalence we observed. A comparison of the MDRD and CKD-EPI equations using the earlier NHANES 1999–2006 data (n=16,032) led to a downward revision of CKD prevalence from 13.1% to 11.5% [26]. The tendency of the CKD-EPI equation to more accurately classify lower-risk patients into higher eGFR categories and result in lower prevalence estimates of CKD has also been documented in a number of other studies [39,40]. However, the most recent report from the US Renal Data System, using the CKD-EPI equation to calculate eGFR, puts the overall prevalence of CKD (including Stage 5 CKD) in the NHANES 2005–2010 population as high as 14.0% [41].

The stratification of CKD into 5 stages according to the presence of persistent kidney damage and/or level of decline in eGFR was previously based on criteria developed in 2002 by the NKF [1] and subsequently endorsed by the Kidney Disease: Improving Global Outcomes (KDIGO) Foundation [42]. However, refinements to the CKD classification system were proposed to provide a more comprehensive description of CKD severity, disease prognosis, and CV risk [43,44]. For example, the high prevalence of Stage 3 CKD [3], and potential differences in CV risk profiles within this stage [8,45], led some experts to suggest subdividing this category into Stage 3a and 3b CKD (eGFR 45–59 and 30–44 mL/min/1.73 m^2^). This analysis of 2001–2010 NHANES data found that the prevalence of CV-related comorbidities and CV risk factors was significantly higher in Stage 3b versus 3a CKD, including an observed ~2-fold increase in the prevalence of CVD and stroke between Stage 3a and 3b, thus supporting this particular revision of the CKD classification system. Indeed, the recently released KDIGO 2012 Clinical Practice Guideline for the Evaluation and Management of Chronic Kidney Disease [2] now includes the subdivision of Stage 3 CKD, as well as the addition of albuminuria stages and diagnosis of CKD by cause, to enable more precise estimations of disease risk and prognosis in patients with CKD.

The additional economic burden associated with CVD in the context of CKD is substantial, and effective CV risk factor modification in patients with CKD has the potential to significantly reduce healthcare costs and improve patient outcomes. In 2008, costs for general (fee-for-service) Medicare patients with both CKD and CVD exceeded $24 billion and accounted for ~12% of overall Medicare expenditures, despite these patients representing only ~5% of the general Medicare population [46]. Patients with CKD and CVD also had per-person, per-month expenditures that were nearly double those of patients with CKD alone ($1,687 versus $888, respectively) [46]. Similar observations have been made within the managed-care setting. An analysis of 13,796 patients with CKD and their matched controls from a large health-maintenance organization demonstrated that the presence of CKD-related comorbidities such as CHD and diabetes almost doubled the total cost of care in these cohorts, and the costs associated with these comorbidities were disproportionally higher in patients with concomitant CKD [47]. A recent review assessing cost effectiveness analyses of a wide range of pharmacological and non-pharmacological interventions for patients with CKD found that a high proportion of the interventions were dominant over the comparator (suggesting both improved outcomes for patients and lower costs for payors), particularly those relating to antihypertensive therapies in patients with CKD Stages 1–4 [48].

### Limitations

This analysis should be interpreted within the context of the following limitations. Drug utilization and disease history in NHANES participants is obtained by self-report and so may be subject to recall bias. Laboratory measurements were performed on single blood and urine specimens, and relied on participants self-reporting an appropriate period of fasting, the absence of which may result in confounding. The use of urinary albumin and creatinine data from a single urine specimen prevented the inclusion of “persistent” albuminuria in the definition of individuals with Stage 1 and Stage 2 CKD. It is important to note that not all NHANES participants receive routine medical care; while this allows for the generalizability of the data to the overall US population, it could be considered a limitation when interpreting lipid and BP treatment and control rates. Finally, caution should be exercised in drawing strong inferences on US population trends based on comparisons between Stage 1 and Stage 4 CKD due to the low number of participants with CKD Stage 4 (n=60) identified from the NHANES 2001–2010 fasting subsample.

## Conclusion

Although significant increases in lipid and BP treatment and control rates were observed over the past decade, these nationally representative trends suggest that continued efforts by healthcare professionals are required to achieve recommended treatment goals for CV risk factors and reduce the economic and social burden of CV complications associated with CKD. Given the observed high prevalence of concomitant CV comorbidities in US adults with advanced CKD, and the potential to reduce future CV events and/or progression of renal disease, traditional CV risk factors such as hypertension and dyslipidemia should be treated more intensively in this population.

## Competing interests

This study was funded by Pfizer Inc. All authors are employees of Pfizer Inc. with ownership of stock in Pfizer Inc.

## Authors’ contributions

AK and LT designed the study and wrote the manuscript. JM performed the data extraction and analyses, and revised the manuscript. All authors read and approved the final manuscript.

## Pre-publication history

The pre-publication history for this paper can be accessed here:

http://www.biomedcentral.com/1471-2369/14/132/prepub

## Supplementary Material

Additional file 1: Table S1 Population characteristics of US adults ≥20 years of age with CKD Stage 1–4* by LDL-C and BP goal attainment status based on NHANES 2001–2010 survey participants. Click here for file
