# Supplementary material for: Evaluation of cardiovascular disease burden and therapeutic goal attainment in US adults with chronic kidney disease: an analysis of national health and nutritional examination survey data, 2001–2010
Source: BMC Nephrol. 2013 Jun 27;14:132. doi: 10.1186/1471-2369-14-132 (PMC3701605; doi:10.1186/1471-2369-14-132)
Supplement: Additional file 1: Table S1 — Population characteristics of US adults ≥20 years of age with CKD Stage 1–4* by LDL-C and BP goal attainment status based on NHANES 2001–2010 survey participants. [file 1471-2369-14-132-S1.pdf]

Additional File 1: Table S1 Population characteristics of US adults ≥20 years of age with CKD Stage 1–4* by LDL-C and BP goal attainment status based on NHANES 2001–2010 survey participants

Characteristic	At LDL-C Goal <100 mg/dL			At BP Goal ≤130/80 mmHg			At Both LDL-C Goal <100 mg/dL and BP Goal ≤130/80 mmHg		
	**Yes (n=567)**	**No (n=861)**	***P*†**	**Yes (n=595)**	**No (n=833)**	***P*†**	**Yes (n=267)**	**No (n=1161)**	***P*†**
Age at screening (years)	65.2 (0.9)	63.5 (0.8)	0.138	60.6 (1.2)	67.0 (0.7)	<0.001	62.1 (1.6)	64.7 (0.7)	0.133
Male (%)	43.4 (2.7)	40.6 (1.6)	0.342	41.0 (2.5)	42.3 (2.2)	0.719	46.0 (3.9)	40.7 (1.6)	0.198
Race/ethnicity (%)			0.665			0.022			0.504
Non-Hispanic white	76.8 (2.2)	74.5 (2.5)		77.7 (2.6)	73.6 (2.4)		77.2 (3.4)	75.0 (2.2)	
Non-Hispanic black	10.8 (1.4)	12.1 (1.3)		9.3 (1.2)	13.4 (1.5)		8.7 (1.7)	12.3 (1.2)	
Mexican American	5.1 (0.9)	6.1 (1.0)		6.8 (1.2)	4.8 (0.9)		6.3 (1.4)	5.6 (0.9)	
Other	7.3 (1.5)	7.2 (1.7)		6.1 (1.5)	8.2 (1.7)		7.8 (2.3)	7.1 (1.6)	
eGFR (mL/min/1.73m^2)	66.1 (1.5)	70.0 (1.3)	0.037	70.7 (1.7)	66.6 (1.1)	0.043	67.1 (2.3)	68.8 (1.1)	0.504
Body mass index (kg/m^2)	30.0 (0.4) (n=543)	29.4 (0.3) (n=829)	0.306	29.0 (0.3) (n=574)	30.2 (0.4) (n=798)	0.021	29.8 (0.5) (n=257)	29.6 (0.3) (n=1115)	0.838
Blood pressure (mmHg)									
Systolic	130.5 (1.4)	135.1 (1.1)	0.015	113.8 (0.6)	148.9 (0.9)	<0.001	112.6 (0.9)	138.2 (0.9)	<0.001
Diastolic	64.4 (0.8)	69.5 (0.7)	<0.001	62.1 (0.9)	71.9 (0.8)	<0.001	59.3 (1.2)	69.5 (0.6)	<0.001
Lipids (mg/dL)									
Total cholesterol	157.5 (1.4)	218.7 (1.5)	<0.001	188.1 (2.2)	199.2 (1.9)	<0.001	155.3 (1.8)	203.7 (1.5)	<0.001

LDL-C	77.3 (1.0)	134.1 (1.1)	<0.001	108.0 (1.9)	114.1 (1.7)	0.036	77.6 (1.3)	119.6 (1.3)	<0.001
HDL-C	53.6 (0.9)	54.9 (0.7)	0.291	53.4 (0.7)	55.1 (0.7)	0.076	52.4 (1.2)	54.8 (0.6)	0.089
Triglycerides	133.2 (4.0)	148.9 (3.2)	0.005	133.5 (3.5)	150.0 (3.3)	0.002	126.6 (5.7)	146.5 (2.8)	0.006
Medication use (%)‡									
Antidiabetics§	32.2 (2.3)	13.5 (1.6)	<0.001	21.2 (1.9)	20.8 (1.8)	0.848	31.8 (3.4)	18.4 (1.5)	<0.001
Antihyperlipidemics‖	40.3 (2.7)	23.8 (1.8)	<0.001	26.8 (2.5)	33.2 (1.9)	0.057	36.0 (3.7)	29.0 (1.5)	0.062
Antihypertensives¶	64.0 (2.8)	48.0 (2.3)	<0.001	46.3 (2.3)	60.9 (2.0)	<0.001	55.6 (4.2)	54.1 (1.9)	0.772
Cardiovascular disease history (%)‡									
Cardiovascular disease#	37.5 (2.5)	22.4 (1.9)	<0.001	29.5 (2.3)	27.6 (1.9)	0.486	41.0 (3.4)	25.4 (1.7)	<0.001
Coronary heart disease**	26.8 (2.3)	14.9 (1.4)	<0.001	20.9 (1.8)	18.6 (1.5)	0.275	30.7 (3.5)	16.9 (1.2)	<0.001
Stroke	11.5 (1.4)	9.5 (1.5)	0.324	9.5 (1.5)	10.9 (1.3)	0.460	9.6 (1.6)	10.5 (1.3)	0.691
Congestive heart failure	14.8 (1.5)	6.3 (1.1)	<0.001	9.8 (1.6)	9.6 (1.3)	0.930	15.3 (2.3)	8.3 (1.0)	0.003
Cardiovascular risk factors (%)‡									
Diabetes (%)††	40.4 (2.6)	25.6 (1.8)	<0.001	28.9 (2.1)	33.7 (2.1)	0.094	40.3 (3.8)	29.4 (1.7)	0.007
Hyperlipidemia (%)‡‡	40.3 (2.7)	63.1 (2.3)	<0.001	45.1 (2.5)	61.0 (2.0)	<0.001	36.0 (3.7)	58.3 (1.7)	<0.001
Hypertension (%)§§	78.3 (2.7)	74.5 (2.0)	0.303	46.3 (2.3)	100.0 (0.0)	NA	55.6 (4.2)	81.0 (1.6)	<0.001

Values are weighted estimates presented as percent (standard error) or mean (standard error). Estimates were standardized to the July 2008 US census population ≥20 years of age. Conversion factors for units: eGFR in mL/min/1.73m^2 to mL/s/1.73m^2, ×0.01667; total cholesterol, LDL-C and HDL-C in mg/dL to mmol/L, ×0.02586; triglycerides in mg/dL to mmol/L, ×0.01129; glucose in mg/dL to mmol/L, ×0.05551. NA, not applicable.

*CKD Stage 1–4 was identified by the presence of kidney damage (based on albuminuria) and level of decline in kidney function (based on eGFR), and staged using modified National Kidney Foundation criteria (see Methods).

†*P* values are for at goal versus not at goal; *P* value for race/ethnicity compares the distribution of race/ethnicity categories. Rao-Scott chi-square *P* values for categorical variables were obtained using the SAS procedure SURVEYFREQ; between-cohort *P* values for continuous variables were obtained using the SAS procedure SURVEYREG (see Methods).

‡All drug utilization and disease history was self-reported.

§Any antidiabetic agents including insulin and oral medications for diabetes.

‖Any lipid-lowering agents including statins, fibric acid derivatives, bile acid sequestrants, cholesterol absorption inhibitors and other antihyperlipidemic agents.

¶Any antihypertensive agents including β-blockers, calcium channel blockers, diuretics, angiotensin-converting enzyme inhibitors, angiotensin receptor blockers and other BP-lowering agents.

#Cardiovascular disease was a composite of self-reported CHD, stroke or CHF.

**Coronary heart disease was identified by self-report of CHD, angina or myocardial infarction.

††Diabetes was identified by self-report, self-reported use of insulin or oral medications for diabetes, or fasting glucose ≥126 mg/dL.

‡‡Hyperlipidemia was defined as LDL-C ≥160 mg/dL for individuals with ≤1 CHD risk factor, ≥130 mg/dL for individuals with ≥2 CHD risk factors, ≥100 mg/dL for individuals with CHD or CHD risk equivalents (eg, diabetes), or self-reported use of lipid-lowering agents.

§§Hypertension was defined as an average BP >130 mmHg systolic or >80 mmHg diastolic, or self-reported use of antihypertensive agents.
